# Point-of-care ultrasound training for residents in anaesthesia and critical care: results of a national survey comparing residents and training program directors’ perspectives

**DOI:** 10.1186/s12909-022-03708-w

**Published:** 2022-08-28

**Authors:** Silvia Mongodi, Francesca Bonomi, Rosanna Vaschetto, Chiara Robba, Giulia Salve, Carlo Alberto Volta, Elena Bignami, Luigi Vetrugno, Francesco Corradi, Salvatore Maurizio Maggiore, Paolo Pelosi, Francesco Mojoli

**Affiliations:** 1 Anesthesia and Intensive Care 1, Fondazione IRCCS Policlinico S. Matteo, Pavia, Italy; 2 Rianimazione I, Fondazione IRCCS Policlinico S. Matteo, DEA piano -1, Viale Golgi 19, 27100 Pavia, Italy; 3 Anesthesia and Intensive Care, ASST-Pavia – Civil Hospital of Vigevano, Pavia, Italy; 4 Department of Translational Medicine, Eastern Piedmont University, Novara, Italy; 5 Anesthesia and Intensive Care, Ospedale Maggiore della Carità, Novara, Italy; 6 Anesthesia and Intensive Care, IRCCS for Oncology and Neurosciences, San Martino Policlinico Hospital, Genoa, Italy; 7 Department of Surgical Sciences and Integrated Diagnostics, University of Genoa, Genoa, Italy; 8 Department of Clinical-Surgical, Diagnostic and Pediatric Sciences, Unit of Anesthesia and Intensive Care, University of Pavia, Pavia, Italy; 9 Department of Morphology, Surgery and Experimental Medicine, University of Ferrara, Ferrara, Italy; 10 Department of Anesthesia and Intensive Care, Parma University Hospital, Parma, Italy; 11 Department of Medical, Oral and Biotechnological Sciences, University of Chieti-Pescara, Chieti, Italy; 12 Department of Anesthesiology, Critical Care Medicine and Emergency, SS. Annunziata Hospital, Chieti, Italy; 13 Anesthesia and Intensive Care, Ente Ospedaliero Ospedali Galliera, Genoa, Italy; 14 Department of Surgical, Medical and Molecular Pathology and Critical Care Medicine, University of Pisa, Pisa, Italy

**Keywords:** Point-of-care ultrasound, Ultrasound education, Training, Residency school organization, Ultrasound curriculum, Teaching

## Abstract

**Background:**

Point-of-care ultrasound (POCUS) has become an essential tool for anaesthesia and critical care physicians and dedicated training is mandatory. This survey describes the current state of Italian residency training programs through the comparison of residents’ and directors’ perspective.

**Methods:**

Observational prospective cross-sectional study: 12-question national e-survey sent to Italian directors of anaesthesia and critical care residency programs (*N* = 40) and residents (*N* = 3000). Questions focused on POCUS teaching (vascular access, transthoracic echocardiography, focused assessment for trauma, transcranial Doppler, regional anaesthesia, lung and diaphragm ultrasound), organization (dedicated hours, teaching tools, mentors), perceived adequacy/importance of the training and limiting factors.

**Results:**

Five hundred seventy-one residents and 22 directors completed the survey. Bedside teaching (59.4–93.2%) and classroom lessons (29.7–54.4%) were the most frequent teaching tools. Directors reported higher participation in research projects (*p* < 0.05 for all techniques but focused assessment for trauma) and simulation (*p* < 0.05 for all techniques but transthoracic echocardiography). Use of online teaching was limited (< 10%); however, 87.4% of residents used additional web-based tools. Consultants were the most frequent mentors, with different perspectives between residents (72.0%) and directors (95.5%; *p* = 0.013). Residents reported self-training more frequently (48.5 vs. 9.1%; *p* < 0.001). Evaluation was mainly performed at the bedside; a certification was not available in most cases (< 10%). Most residents perceived POCUS techniques as extremely important. Residents underestimated the relevance given by directors to ultrasound skills in their evaluation and the minimal number of exams required to achieve basic competency. Overall, the training was considered adequate for vascular access only (62.2%). Directors mainly agreed on the need of ultrasound teaching improvement in all fields. Main limitations were the absence of a standardized curriculum for residents and limited mentors’ time/expertise for directors.

**Conclusion:**

POCUS education is present in Italian anaesthesia and critical care residency programs, although with potential for improvement. Significant discrepancies between residents’ and directors’ perspectives were identified.

**Supplementary Information:**

The online version contains supplementary material available at 10.1186/s12909-022-03708-w.

## Background

Ultrasound is a bedside non-irradiating tool and is now easily available in hand-held devices; it allows integrative head-to-toe clinical assessment as well as guidance for invasive procedures. For these reasons, ultrasound has recently become ever more present in the hands of anaesthesia and critical care physicians [1]. Anaesthesiologists and intensivists’ ultrasound skills started with intraoperative transoesophageal echocardiography [2], but rapidly spread to vascular access [3] and regional anaesthesia [4]. In critical care, the last few decades showed an increase in point-of-care ultrasound (POCUS) [5] for hemodynamic [6], respiratory [7, 8] and neurologic assessment [9]. POCUS has also become helpful for assessing trauma patients [10], making a differential diagnosis in acute respiratory failure [11], redirecting treatment [12] and replacing traditional imaging [13, 14]. Each ultrasound technique requires adequate training, since POCUS can be misleading when performed by inexperienced operators [15]. Skill levels and corresponding minimum requirements for training have been established for brain [16], lung [17, 18] and cardiac [19] ultrasound and for ultrasound-guided procedures [20]; this was the starting point to define dedicated training pathways for intensivists and anaesthesiologists [21, 22]. However, recent studies showed remarkable heterogenicity in ultrasound training programs all around the world [23–30] and the need for a standardized ultrasound training program remains a relevant issue [24, 31]. The purpose of this survey was to describe the current state and limitations of ultrasound training in Italian anaesthesia and critical care residency programs; the identification of weaknesses and strengths from two different points of view (training program directors and residents) was considered a first step to improve the education system and to structure a national ultrasound curriculum for intensivists and anaesthesiologists.

## Methods

This is an observational prospective cross-sectional study: following accepted research practices for surveys [32, 33], we conducted a closed e-survey on the ultrasound training programs for vascular access (VA), lung ultrasound (LUS), transthoracic echocardiography (TTE), focused-assessment for trauma (FAST), transcranial Doppler (TCD), regional anaesthesia (RA) and diaphragm ultrasound (DUS) during the 5-year residency school in anaesthesia and critical care residency schools in Italy. Residency schools in Italy are university entities responsible for teaching and training medical residents. The survey included questions on the teaching organization (number of hours for theoretical training, teaching tools, availability of tutors devolved to each ultrasound field), perceived adequacy and importance of the training, limiting factors and potential improvements. The ethical committee (Comitato Etico Pavia) of the Fondazione IRCCS Policlinico S. Matteo waived the need for ethics approval and consent to participate. The study was approved and supported by the College of Professors in Anaesthesiology and Critical Care (CPAR).

### Sampling

The same 12-question e-survey was sent to two target populations via e-mail by the Italian CPAR to recruit directors of residency programs (*N* = 40) who were then asked to send the survey link to their residents (estimated number = 3000). Five more questions were added to residents’ survey to investigate their use of additional learning tools. The survey remained accessible from October 2018 until December 2019; once sent, the responders were not able to review and change their answers. Data were not stored if the survey was not completed (participation rate = completion rate).

### Questionnaire

The survey included open and closed questions (both multiple choice and Likert-like questions – e-Appendix 1 and 2); it was implemented using a Google form which provided an intuitive interface and automatic data export. Adequacy of contents, correct functioning of the form, and quick filling time (less than ten minutes) were tested on a sample of 20 students before the beginning of the study. Residents’ responses were collected anonymously; though the survey asked which school the residents and directors belonged to, as a way to analyse geographic distribution, facilitate personalized follow-up calls and identify duplicates, this information was not further analysed. Follow-up e-mails were sent 4 times to directors, while no direct contact was available with residents. There were no incentives for participation.

### Statistical analysis

Data are displayed as numbers and percentages. Comparisons between directors’ and residents’ answers were performed by Fisher’s exact test. Comparisons excluded answers like “I don’t know” / “not yet encountered in my training path” since expected in residents’ answers only. Analysis was performed by STATA SE 14 for Macintosh.

## Results

### Sample of survey respondents

We obtained 22/40 answers from directors (26 actual answers, 4 duplicates, response rate 55.0%); 3/6 were from Southern Italy, 3/8 from Central Italy, 14/18 from Northern Italy, 2/5 from the islands. 571 residents from 30 residency schools filled in the survey (75.0% of residency schools represented, overall response rate 19.0%); 95 (16.6%) were from Southern Italy, 54 (9.5%) from Central Italy, 410 (71.8%) from Northern Italy and 12 (2.1%) from the islands. Northern Italy resulted to be more represented; however, it also holds 45.0% of the residency schools. Residents were homogeneously distributed among the five years of residency school (first 18.0%, second 20.1%, third 21.4%, fourth 24.2%, fifth 16.3%). 21 schools were represented by both residents and directors; in 1 case, we only received director’s answer. In a minority of cases, schools were represented by residents only (65 residents, 10 schools – e-Fig. 1).

### Teaching organization

**Fig. 1:**
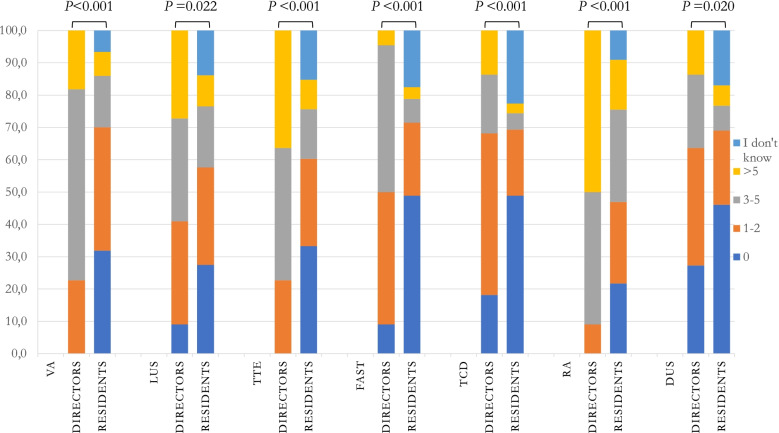
Hours dedicated to each technique along the 5 years of residency school according to directors and residents. VA: vascular access; LUS: lung ultrasound; TTE: transthoracic echocardiography; FAST: focused assessment with sonography in trauma; TCD: transcranial Doppler; RA: regional anaesthesia; DUS: diaphragm ultrasound. The comparison excluded those answering: “I don’t know”, being expected among residents only

The teaching tools used for ultrasound training are displayed in Table 1. Bedside teaching was the most frequently used for all ultrasound techniques. A significant difference between residents’ and directors’ point of view was observed for FAST (57.9 vs. 86.4%; *p* = 0.007) and DUS (59.4 vs. 81.8%; *p* = 0.043). The second most frequently used tool was classroom lessons (*i.e*., teacher-centred instruction taking place from the front of the classroom) but with lectures reported as more frequent and longer in hours by directors for all the ultrasound techniques (Fig. 1). A higher participation in research projects was reported by directors for all techniques but FAST (*p* < 0.005). Simulation was not frequently used, yet with a different perception by residents and directors for VA (19.2 vs. 45.5%; *p* = 0.006), FAST (9.6 vs. 27.3%; *p* = 0.019) and TCD (5.3 vs. 18.2%; *p* = 0.035). Online modules were used in < 10% of cases, according to both directors and residents. Residents reported the use of additional learning tools like web-based teaching (webinars, tutorials, videos – 87.4%), books and scientific literature (79.9%), extra-curricular courses (52.4%) and others (5.3%). According to residents, 55.9% attended an extra-curricular ultrasound course (*i.e*., a course external to the residency school requiring financial support); 14.5% were sponsored by the residency school. 77.3% of directors reported to have supported at least 25% of residents for an extra-curricular ultrasound course. Consultant physicians were the most frequent mentors, however with a significantly different perception (residents’ point of view: 72.0 vs. directors’ point of view: 95.5%; *p* = 0.013 – e-Fig. 2). Residents reported self-training as more frequent (48.5 vs. 9.1%; *p* < 0.001). In 12.3 (VA) to 29.6% (TCD) of cases, residents reported there was no minimum declared number of exams required to achieve basic competency, while directors considered adequate for a resident’s training a minimum number of 1–10 exams for each technique (e-Fig. 3). The assessment of ultrasound competency was described as mainly performed at the bedside by both directors (68.2%) and residents (58.2%; *p* = 0.373, e-Fig. 4); residents reported a higher frequency of no assessment (37.7 vs. 9.1%; *p* = 0.06) and a lower one of theoretical examinations (12.2 vs. 45.5%; *p* < 0.001). Formal certification of theoretical and practical competency was performed in a minority of cases from the point of view of both residents and directors (7.7 and 9.1%; *p* = 0.685, e-Fig. 4). Ultrasound machines were mostly available, mainly in the ICU (e-Table 1); pre-hospital medicine resulted to be the less equipped setting according to both directors and residents.

**Table 1:** Teaching tools adopted for different ultrasound techniques

	**Bedside teaching**			**Online modules**			**Classroom lessons**			**Simulation**			**Research**			**None**			**Not yet** encountered **in my training**
	**R**	**D**	***P* value**	**R**	**D**	***P* value**	**R**	**D**	***P* value**	**R**	**D**	***P* value**	**R**	**D**	***P* value**	**R**	**D**	***P* value**	**R**
VA	520 (93.2)	21 (95.5)	1.000	22 (3.9)	2 (9.1)	0.230	204 (36.6)	18 (81.8)	**< 0.001**	107 (19.2)	10 (45.5)	**0.006**	34 (6.1)	7 (31.8)	**< 0.001**	19 (3.49)	0 (0.0)	1.000	13
LUS	426 (84.4)	18 (81.8)	0.764	36 (7.1)	0 (0.0)	0.388	232 (45.9)	15 (68.2)	**0.049**	67 (13.3)	6 (27.3)	0.104	88 (17.4)	11 (50.0)	**0.001**	29 (5.7)	1 (4.5)	1.000	66
TTE	356 (74.3)	20 (90.9)	0.127	27 (5.6)	0 (0.0)	0.623	205 (42.8)	14 (63.6)	0.077	43 (9.0)	4 (18.2)	0.141	40 (8.4)	5 (22.7)	**0.038**	60 (12.5)	0 (0.0)	**0.097**	92
FAST	259 (57.9)	19 (86.4)	**0.007**	25 (5.6)	0 (0.0)	0.621	130 (29.1)	15 (68.2)	**< 0.001**	43 (9.6)	6 (27.3)	**0.019**	11 (2.5)	2 (9.1)	0.120	119 (26.6)	1 (4.5)	**0.022**	124
TCD	256 (61.8)	15 (68.2)	0.655	27 (6.5)	0 (0.0)	0.384	123 (29.7)	14 (63.6)	**0.002**	22 (5.3)	4 (18.2)	**0.035**	33 (8.0)	6 (27.3)	**0.009**	102 (24.6)	3 (13.6)	0.312	157
RA	459 (86.4)	22 (100)	0.097	36 (6.8)	1 (4.5)	1.000	289 (54.4)	19 (86.4)	**0.003**	87 (16.4)	5 (22.7)	0.390	35 (6.6)	7 (31.8)	**0.001**	25 (4.7)	0 (0.0)	0.616	40
DUS	266 (59.4)	18 (81.8)	**0.043**	31 (6.9)	1 (4.5)	1.000	151 (33.7)	14 (63.6)	**0.006**	39 (5.7)	3 (13.6)	0.434	92 (20.5)	14 (63.6)	**< 0.001**	108 (24.1)	3 (13.6)	0.315	123

Data are displayed as values (percentage). In bold: statistically significant p values for comparison between residents and directors for each ultrasound technique and teaching tool; Fisher exact and percentages were computed excluding those answering: “Not yet encountered in my training” in residents’ answers

*R* Residents, *D* Directors, *VA* Vascular access, *LUS* Lung ultrasound, *TTE* Transthoracic echocardiography, *FAST* Focused assessment with sonography in trauma, *TCD* Transcranial Doppler, *RA* Regional anaesthesia, *DUS* Diaphragm ultrasound

### Perceived importance and adequacy of training

**Fig. 2:**
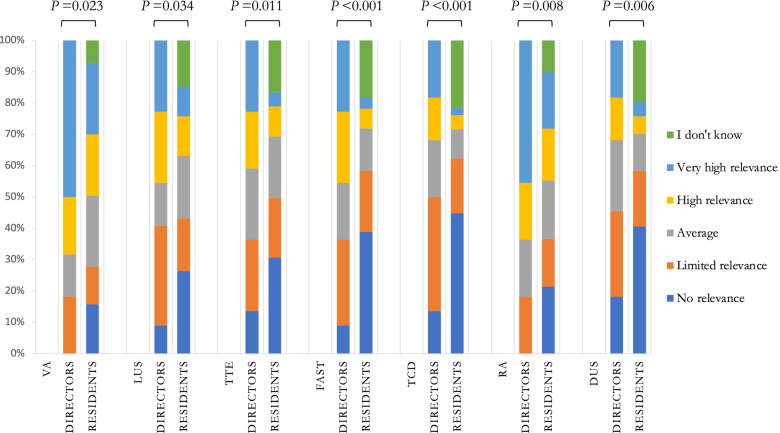
Relevance of ultrasound competences in the global evaluation of the residents according to directors and residents. VA: vascular access; LUS: lung ultrasound; TTE: transthoracic echocardiography; FAST: focused assessment with sonography in trauma; TCD: transcranial Doppler; RA: regional anaesthesia; DUS: diaphragm ultrasound

The impact of ultrasound competency on clinical activity was mainly perceived by residents as extremely important (e-Fig. 5), for procedural safety (VA 96.3% and RA 89.6%) and for providing additional clinical information (LUS 89.3%, TTE 88.8%, TCD 72.7%, FAST 84.4%, DUS 69.9%—e-Fig. 6). The relevance of ultrasound competency in the global evaluation of the residents is shown in Fig. 2; residents tend to underestimate the relevance given by directors. The training was described by residents mainly as adequate or more than adequate for VA (62.2%) and RA (46.1%) and mainly as inadequate or very inadequate for all the other techniques (FAST 54.6%, TCD 50.3%, TTE 46.4%, DUS 45.2%, LUS 33.1%—e-Fig. 7). Accordingly, residents felt mostly confident or very confident in VA only (58.7%), while they felt uncomfortable or very uncomfortable in practicing all the other techniques (TCD 87.6%, DUS 80.6%, FAST 76.2%, TTE 75.5%, LUS 50.4%, RA 48.0%—e-Fig. 8). Directors mainly agreed or strongly agreed on the need of ultrasound training improvement in all the analysed fields (TTE 54.5%, FAST 50.0%, LUS 45.5%, TCD 40.9%, RA 40.9% DUS 36.4%—e-Fig. 9) except for VA (18.2%).

### Limiting factors and potential improvement

Limiting factors are displayed in Table 2. From the residents’ point of view, the most relevant limiting factor for all the analysed techniques was the lack of a standardized training program (VA 48.7%, LUS 49.9%, TTE 52.7%, FAST 52.5%, TCD: 45.5%, RA: 40.6%, DUS: 43.4%), followed by limited availability and skills from mentors. According to the directors’ opinion, limited mentors’ skills were the most relevant limiting factor for most of the techniques.

**Table 2:** Limiting factors for ultrasound training

	**Limited mentor’s time-availability**			**Limited mentor’s skills**			**Ultrasound machine availability**			**Limited resident’s time-availability**			**Lack of a standardized training program**			**I don’t know**	**No clear limitations**
	**R**	**D**	***P* value**	**R**	**D**	***P* value**	**R**	**D**	***P* value**	**R**	**D**	***P* value**	**R**	**D**	***P* value**	**R**	**D**
VA	209 (38.2)	4 (18.2)	0.057	72 (26.0)	3 (13.6)	0.949	165 (30.2)	8 (36.4)	**0.535**	100 (18.3)	0 (0.0)	**0.027**	278 (50.8)	7 (31.8)	**0.080**	24 (4.4)	8 (36.4)
LUS	191 (38.7)	8 (36.4)	0.823	147 (32.9)	10 (45.5)	0.119	108 (21.9)	5 (22.7)	**0.927**	80 (16.2)	0 (0.0)	0.040	285 (57.8)	7 (31.8)	**0.016**	78 (14.3)	3 (13.6)
TTE	208 (43.4)	7 (31.8)	0.282	164 (34.2)	7 (31.8)	0.815	106 (22.1)	4 (18.2)	**0.662**	85 (17.7)	2 (9.1)	0.295	301 (62.8)	7 (31.8)	**0.003**	92 (16.8)	2 (9.1)
FAST	159 (34.3)	6 (27.3)	0.498	142 (30.6)	8 (36.4)	0.568	73 (15.7)	6 (27.3)	**0.152**	67 (14.4)	1 (4.5)	**0.191**	300 (64.7)	9 (40.9)	0.024	107 (19.6)	4 (18.2)
TCD	133 (31.7)	7 (31.8)	0.994	162 (38.7)	9 (40.9)	0.833	76 (18.1)	5 (22.7)	**0.588**	56 (13.4)	1 (4.5)	**0.229**	260 (62.1)	7 (31.8)	**0.050**	152 (27.8)	3 (13.6)
RA	194 (37.5)	6 (27.3)	0.333	154 (29.7)	4 (18.2)	0.244	158 (30.5)	6 (27.3)	**0.747**	99 (19.1)	2 (9.1)	0.238	232 (44.8)	4 (18.2)	**0.014**	53 (9.7)	8 (36.4)
DUS	139 (32.0)	6 (27.3)	0.640	164 (37.8)	9 (40.9)	0.621	75 (17.3)	4 (18.2)	**0.812**	57 (13.1)	0 (0.0)	0.040	247 (56.9)	5 (22.7)	**0.035**	137 (25.0)	5 (22.7)

Data are displayed as values (percentage). In bold: statistically significant p values for comparison between residents and directors, for each technique and limiting factor

R *Residents*, *D* Directors, *VA* Vascular access, *LUS* Lung ultrasound, *TTE* Transthoracic echocardiography, *FAST* Focused assessment with sonography in trauma, *TCD* Transcranial Doppler, *RA* Regional anaesthesia, *DUS* Diaphragm ultrasound

## Discussion

In this survey on the current state of ultrasound training in Italian critical care, anaesthesia, and pain therapy residency schools we found that 1. ultrasound teaching in Italian residency school is mainly based on bedside teaching and classroom lessons, is mentored by consultant physicians, and is perceived as adequate for vascular access only; 2. there are significant discrepancies in residents’ and directors’ perception of many aspects of the training; 3. despite the high relevance of ultrasound competency from both residents and directors, a formal certification of theoretical and practical skills is rarely performed, which is perceived as the main limitation to ultrasound teaching by residents.

The strengths of the present survey are that this is the first prospective survey in Italy for ultrasound training in critical care, anaesthesia, and pain therapy residency schools. Secondly, it clearly focused on questions concerning a variety of aspects of the POCUS training. Finally, it provided opinions from directors and residents for comparison, an essential aspect to improve the education system.

A consensus of the European Society of Intensive Care Medicine recently defined the basic ultrasound knowledge required for all intensivists [34]: most of the ultrasound techniques investigated in this survey are now considered essential for physician in this field and the question on how to structure a shared ultrasound curriculum to effectively acquire and maintain ultrasound expertise is crucial.

Our results show that the most common training tools are bedside teaching and classroom lessons; this is consistent with literature describing them as the easiest and most well-established tools [24]. Recent studies suggested to implement the currently diffuse face-to-face lecture model with the adoption of flipped classroom [35–39], social media [40–43] and online learning [44–48]. Online modules were rarely adopted in Italian training programs, although appreciated by residents who reported an extensive use of web-based tools. It has to be noted that the survey ended before the novel coronavirus 2019 pandemic, that pushed many universities to implement web-based training, hybrid web-based / in-person training and also mobile applications for informal group case-based discussions [49, 50]. Participation in research projects is also an opportunity for young physicians to work with experts in a field, to study a topic in depth and to receive dedicated training; however, this is reported as infrequent by residents. Simulation was infrequent in Italian residency schools, similar to what was previously reported in the United States [24], although it has been shown to enhance knowledge level, dexterity and confidence [51, 52]. Liberal practice should also be encouraged and structured since it is fundamental to improve technical skills and confidence [53].

Residents are mainly mentored by consultant physicians, similar to what has been previously reported in other countries [30, 54]; this implies a potentially ununiform training. POCUS is used and established in different ways and settings on the basis of each hospital’s experience; especially when the most innovative techniques are being used and taught, the expertise is not homogeneous [23, 53, 55]. Such heterogeny may lead to the development of a dysfunctional cycle where consultants who have insufficient expertise [30] are in charge of educating trainees who then perceive their education as inadequate [54]. Accordingly, mentors with limited skills are perceived as the main limitation by directors. Similar barriers to ultrasound training are perceived in other countries [23–25, 29, 30], in particular, the lack of trainers’ expertise and available time and the need for a standardized curriculum. Other core elements have also been suggested to improve ultrasound training, such as structured image storage, documentation, and quality assurance [29].

Overall, the training is perceived as adequate by both residents and directors for VA only; this may be explained by the fact that this basic technique is widely spread among intensivists and anaesthesiologists and corresponds to a training target beginning in the first year of school for all residents. The most neglected technique is FAST, probably because it is mainly performed in extra-hospital scenarios or in the emergency department. In our data, its teaching is nevertheless considered important and could easily be implemented using healthy volunteers with a steep learning curve [56].

Many discrepancies emerged between the opinions of residents and directors. The relevance given to ultrasound competency is high for both residents and directors; however, the relevance given by directors is frequently underestimated by residents. Regarding teaching organization, directors report more classroom lessons, participation to research projects and supported extra-curricular ultrasound courses. The number of required exams to achieve basic competency for each technique is also higher from the directors’ point of view, but not always in line with the literature [16–20]. This discrepancy may be due to difficulties faced by directors in keeping the level of didactic activity and in keeping resident evaluation as high as planned. In addition, a lack of clear communication between directors and residents may lead to the residents underestimating the teaching opportunities offered by the residency schools.

Directors also report a higher percentage of theoretical evaluation of ultrasound competency, probably also considering the assessment of ultrasound competency performed during the general annual residency final examination. Residents seem to prefer a more dedicated and planned training curriculum with a declared number of expected exams and a formal certification, *i.e.,* a shared ultrasound curriculum.

A formal certification is in fact recognized as lacking by both directors and residents, with both groups wishing to improve the quality of ultrasound teaching. The lack of a standardized teaching program is not new in ultrasound training, where national and international societies are trying to set standards for each technique and for a sharable curriculum for ultrasound in critical care [16, 21, 34, 57–60] Some years ago, Galarza et al. [23] compared the state of critical care ultrasound training among European countries: only 5/42 countries had a national training program, and no agreement was found between these five. To investigate the state of ultrasound training in pulmonary critical care fellows in the United States, Brady et al. also sent a survey to program directors, who were then charged with enrolling their fellows [24]: results showed that most of the fellows received some type of formal training and were mainly self-trained at the bedside, while a minority used simulations or could be supervised by a mentor. Mosier et al. [30] described bedside ultrasound use and training among critical care training programs in the United States with a cross-sectional survey sent to program directors: the use and acknowledged usefulness of ultrasound techniques were very high, but directors recognized the need to improve ultrasound training that was mainly based on informal teaching with limited use of simulations, review sessions and dedicated mentors. Mizubuti et al. [25] analyzed 17 Canadian residency training programs for anesthesiologists: formal rotations resulted to be more frequent than what was reported by our survey; however, a well-defined minimum target of exams was set in only 4 training programs. Moreover, it must be noted that the questionnaire was sent to directors only.

To improve ultrasound teaching in anaesthesia and critical care residency schools, based on our findings and previous literature, we suggest: 1. to improve communication between directors and residents via mailing lists, websites and digital reminders to overcome part of the discrepancies between the two groups; 2. to implement those educational approaches that are now used in a limited manner (new technology for online learning, near-peer education, simulation); 3. to structure a standardized training program, with dedicated mentors, well-defined training goals and formal certification, all reported as major limitations by residents; 4. to build an educational network between schools based on ultrasound competency to overcome the lack of expertise in trainers, a major limitation reported by directors.

This survey presents many limitations. First, a lower-than-expected number of responses was obtained, thus the results may not perfectly reflect the state of ultrasound training in Italy; however, the absolute number of participants is high, and the residents’ actual response rate is unknown, since we relied on individual program directors to forward our survey on to their respective residents. Nevertheless, we have a good homogeneity of responses per residency year and geographical distribution. Results were not adjusted as a function of the level of training of the residents; this limitation was mitigated by the possibility to answer “not yet encountered in my training” in each question. Second, the two populations we compared are necessarily very different in numbers of components. Finally, results were not normalized per school, being each composed by a highly variable number of residents, in order not to penalize those with a limited number of responders.

## Conclusions

POCUS education is present in Italian anaesthesia and critical care residency schools, but it does not fulfil the expectations in modalities outside of vascular accesses; the analysis of significant discrepancies between the perspectives of residents and directors may lead to suggestions for improvement of the educational system. Further research is needed to properly plan formal training programs.

## Supplementary Information

**Additional file 1: e-Figure 1.** Bubble chart for the number of residents’ answers in each school according to director answer. In most cases, residents and directors who answered to the survey belonged to the same school (green bubbles). In orange, a minority of schools represented by residents only. Residency schools are shown as progressive number to keep them anonymous.

**Additional file 2: e-Figure 2.** Mentors for ultrasound training as perceived by residents and directors.

**Additional file 3: e-Figure 3.** Number of exams required by the residency school according to residents and considered adequate for training by residency school directors. VA: vascular access; LUS: lung ultrasound; TTE: transthoracic echocardiography; FAST: focused assessment with sonography in trauma; TCD: transcranial Doppler; RA: regional anaesthesia; DUS: diaphragm ultrasound. The comparison excluded those answering: ”I don’t know”, being expected among residents only.

**Additional file 4: e-Figure 4.** Assessment of ultrasound competencies during residency school as perceived by residents and directors.

**Additional file 5: e-Figure 5.** Impact of ultrasound competencies on future working activity by residents. VA: vascular access; LUS: lung ultrasound; TTE: transthoracic echocardiography; FAST: focused assessment with sonography in trauma; TCD: transcranial Doppler; RA: regional anaesthesia; DUS: diaphragm ultrasound.

**Additional file 6: e-Figure 6.** Expected additional value in clinical activity of ultrasound techniques in the residents’ view. VA: vascular access; LUS: lung ultrasound; TTE: transthoracic echocardiography; FAST: focused assessment with sonography in trauma; TCD: transcranial Doppler; RA: regional anaesthesia; DUS: diaphragm ultrasound.

**Additional file 7: e-Figure 7.** Adequacy of ultrasound training as perceived by residents in the different ultrasound techniques. VA: vascular access; LUS: lung ultrasound; TTE: transthoracic echocardiography; FAST: focused assessment with sonography in trauma; TCD: transcranial Doppler; RA: regional anaesthesia; DUS: diaphragm ultrasound.

**Additional file 8: e-Figure 8.** Confidence in performing ultrasound examination and procedures as perceived by residents. VA: vascular access; LUS: lung ultrasound; TTE: transthoracic echocardiography; FAST: focused assessment with sonography in trauma; TCD: transcranial Doppler; RA: regional anaesthesia; DUS: diaphragm ultrasound.

**Additional file 9: e-Figure 9.** Need of improvement in ultrasound teaching in their own residency school as perceived by directors. VA: vascular access; LUS: lung ultrasound; TTE: transthoracic echocardiography; FAST: focused assessment with sonography in trauma; TCD: transcranial Doppler; RA: regional anaesthesia; DUS: diaphragm ultrasound.

**Additional file 10: Appendix 1.** Survey sent to directors.

**Additional file 11: Appendix 2.** Survey sent to residents.

**Additional file 12: e-Table 1.** Availability of ultrasound machines in different clinical contexts according to directors and residents.

## Data Availability

The datasets used and/or analysed during the current study are available from the corresponding author on reasonable request.

## Abbreviations

DUS: Diaphragm ultrasound
FAST: Focused assessment for trauma
LUS: Lung ultrasound
POCUS: Point-of-care ultrasound
RA: Regional anesthesia
TTE: Transthoracic echocardiography
TCD: Transcranial Doppler
VA: Vascular access
