# Supplementary figures and images for: Point-of-care ultrasound training for residents in anaesthesia and critical care: results of a national survey comparing residents and training program directors’ perspectives
Source: BMC Med Educ. 2022 Aug 28;22:647. doi: 10.1186/s12909-022-03708-w (PMC9420188; doi:10.1186/s12909-022-03708-w)

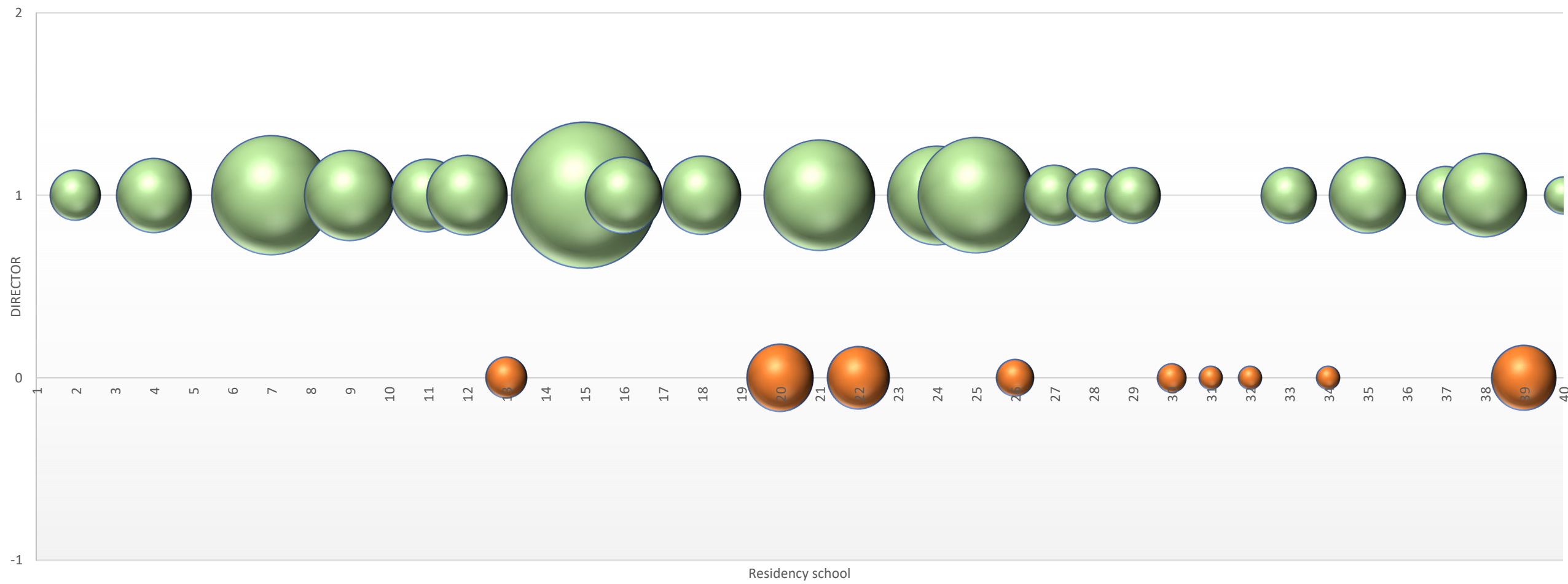

Supplement: Supplementary file 1 — Additional file 1: e-Figure 1. Bubble chart for the number of residents’ answers in each school according to director answer. In most cases, residents and directors who answered to the survey belonged to the same school (green bubbles). In orange, a minority of schools represented by residents only. Residency schools are shown as progressive number to keep them anonymous. [file 12909_2022_3708_MOESM1_ESM.pdf]

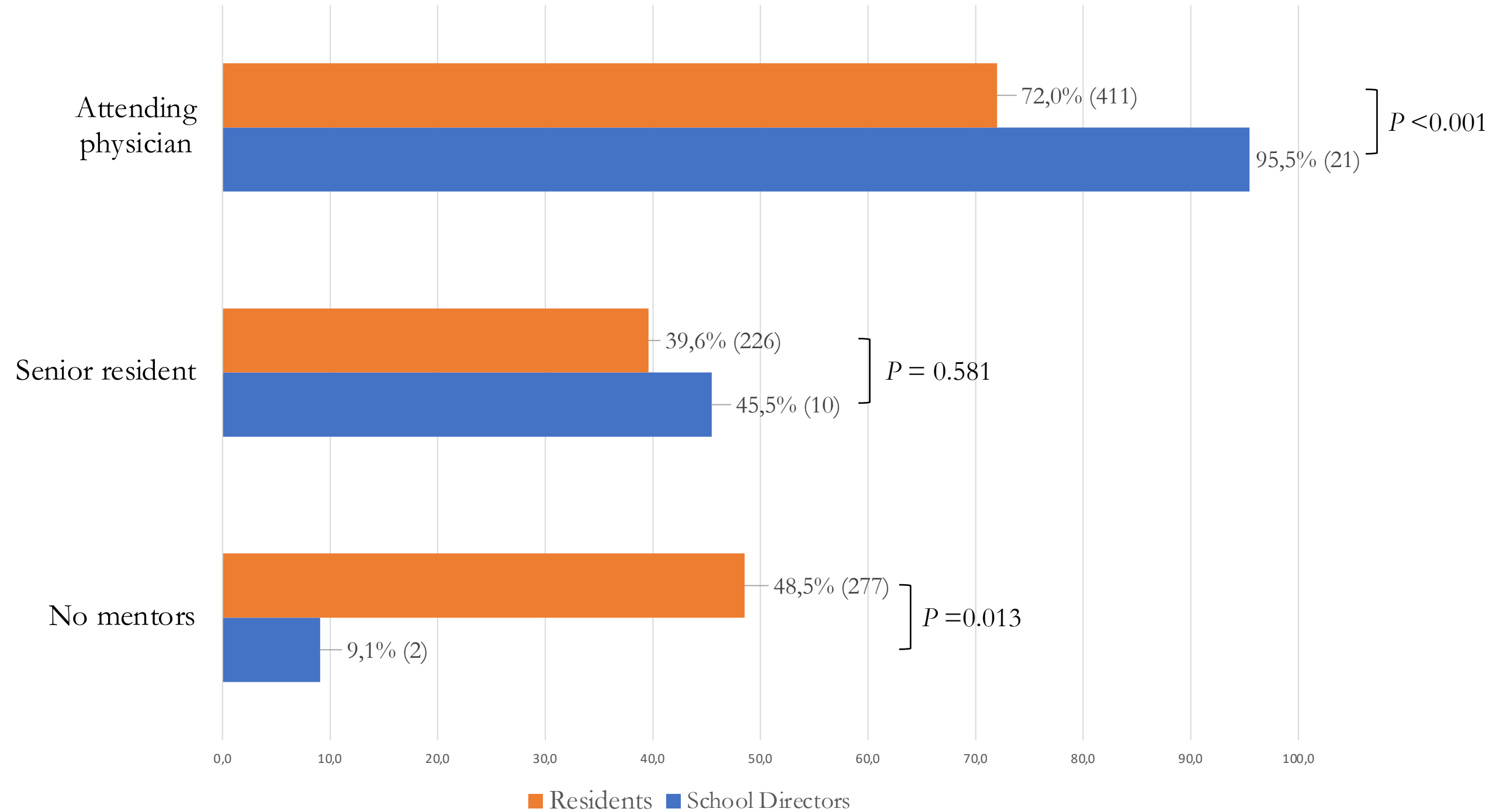

Supplement: Supplementary file 2 — Additional file 2: e-Figure 2. Mentors for ultrasound training as perceived by residents and directors. [file 12909_2022_3708_MOESM2_ESM.pdf]

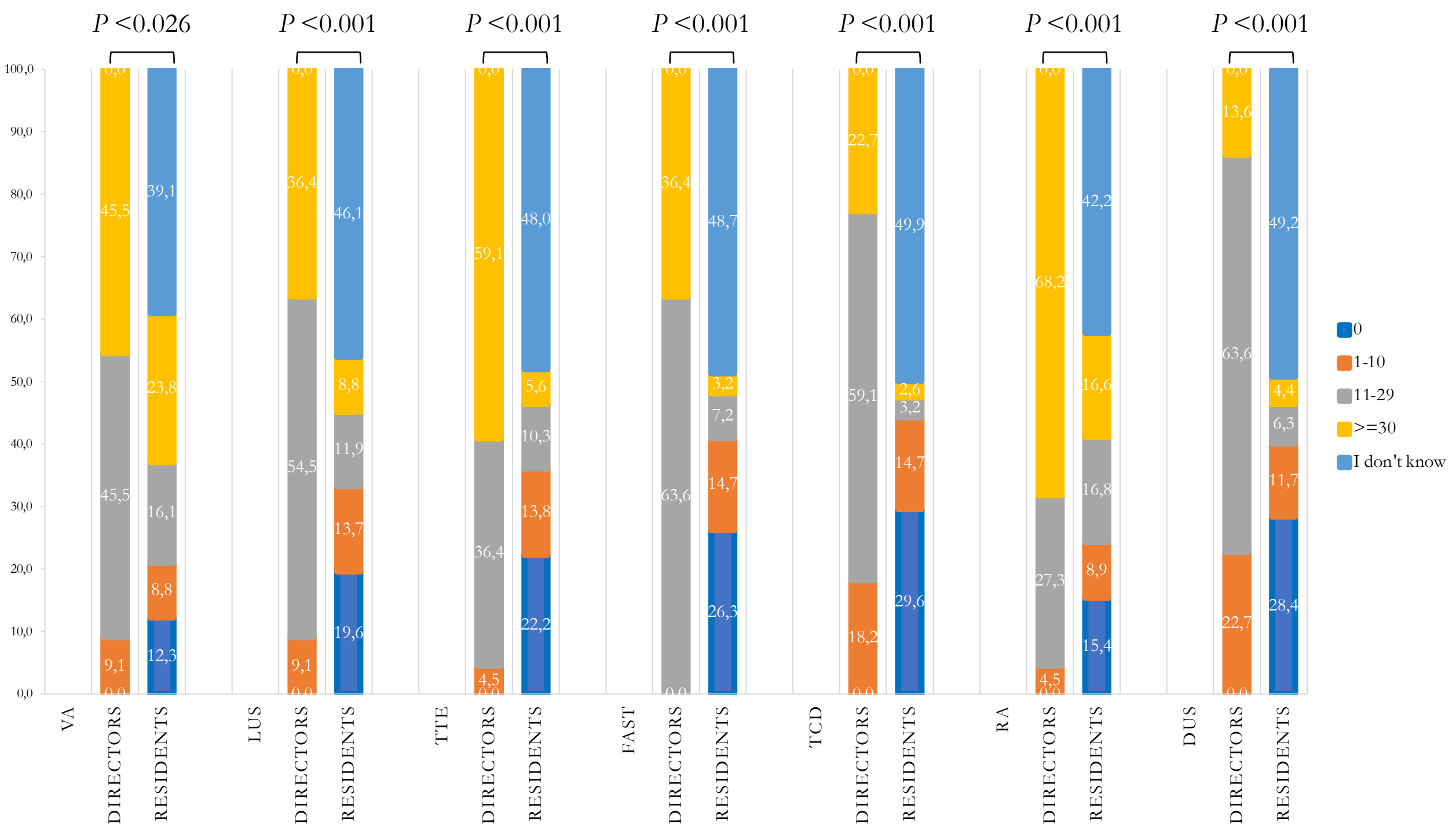

Supplement: Supplementary file 3 — Additional file 3: e-Figure 3. Number of exams required by the residency school according to residents and considered adequate for training by residency school directors. VA: vascular access; LUS: lung ultrasound; TTE: transthoracic echocardiography; FAST: focused assessment with sonography in trauma; TCD: transcranial Doppler; RA: regional anaesthesia; DUS: diaphragm ultrasound. The comparison excluded those answering: ”I don’t know”, being expected among residents only. [file 12909_2022_3708_MOESM3_ESM.pdf]

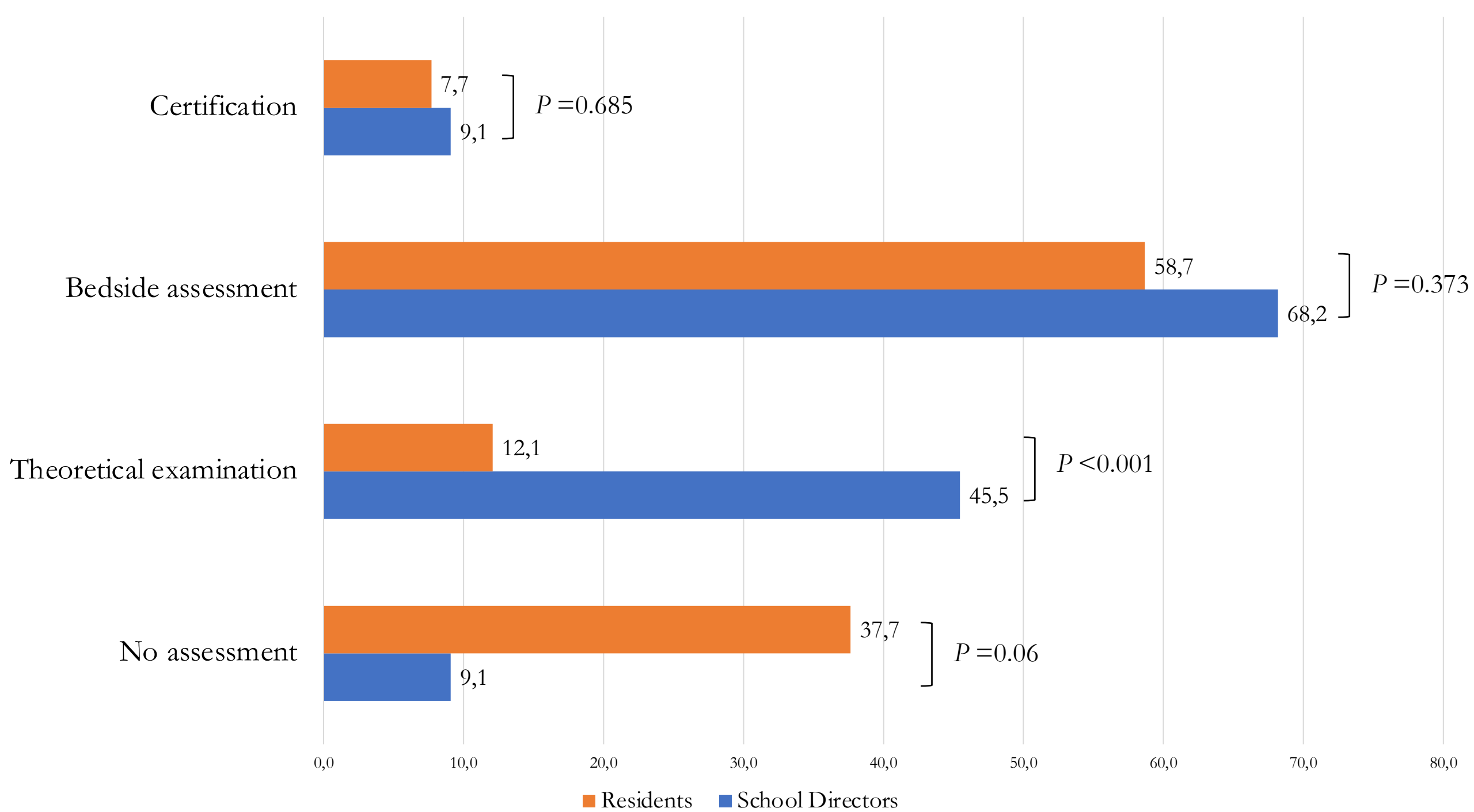

Supplement: Supplementary file 4 — Additional file 4: e-Figure 4. Assessment of ultrasound competencies during residency school as perceived by residents and directors. [file 12909_2022_3708_MOESM4_ESM.pdf]

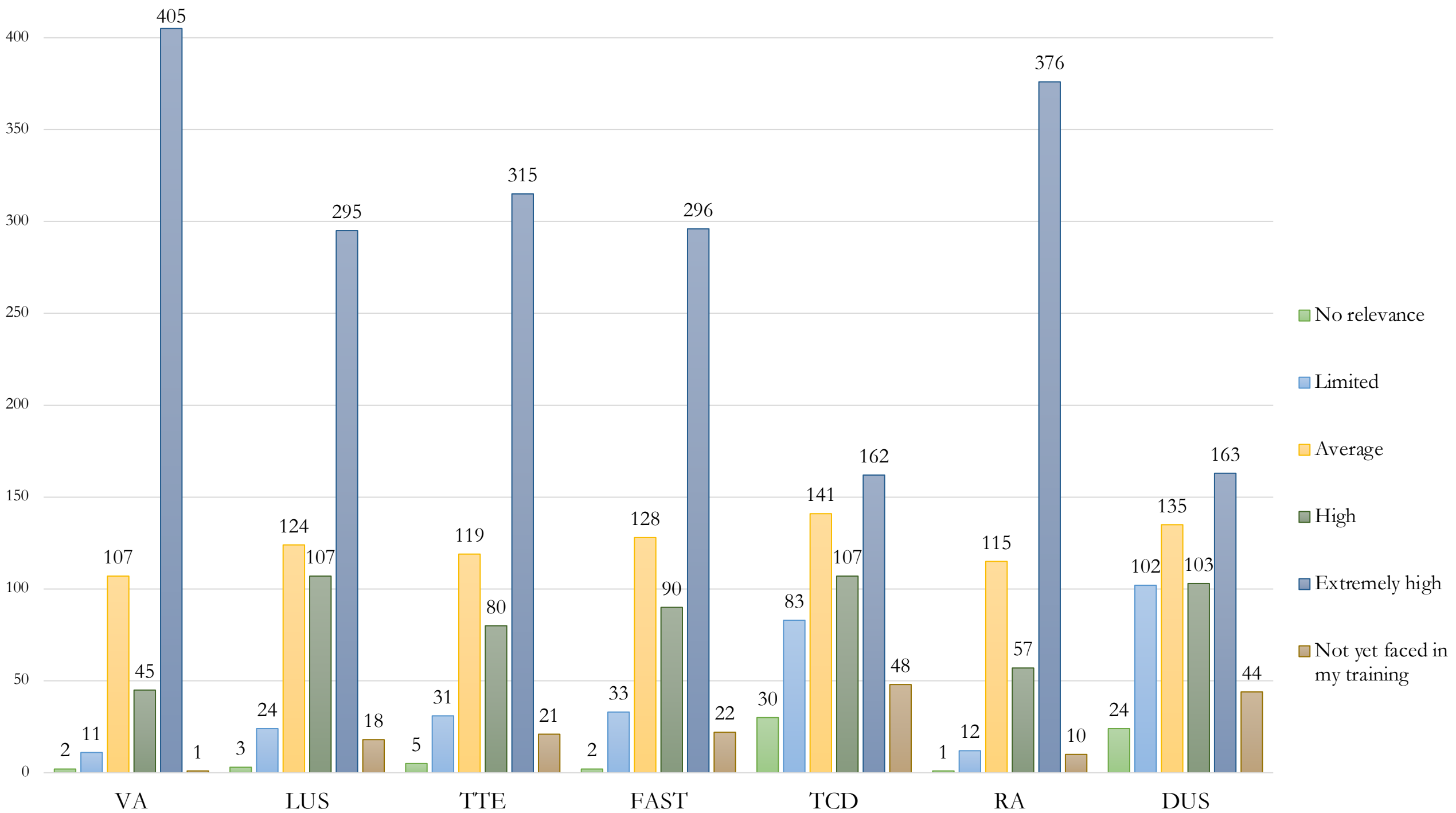

Supplement: Supplementary file 5 — Additional file 5: e-Figure 5. Impact of ultrasound competencies on future working activity by residents. VA: vascular access; LUS: lung ultrasound; TTE: transthoracic echocardiography; FAST: focused assessment with sonography in trauma; TCD: transcranial Doppler; RA: regional anaesthesia; DUS: diaphragm ultrasound. [file 12909_2022_3708_MOESM5_ESM.pdf]

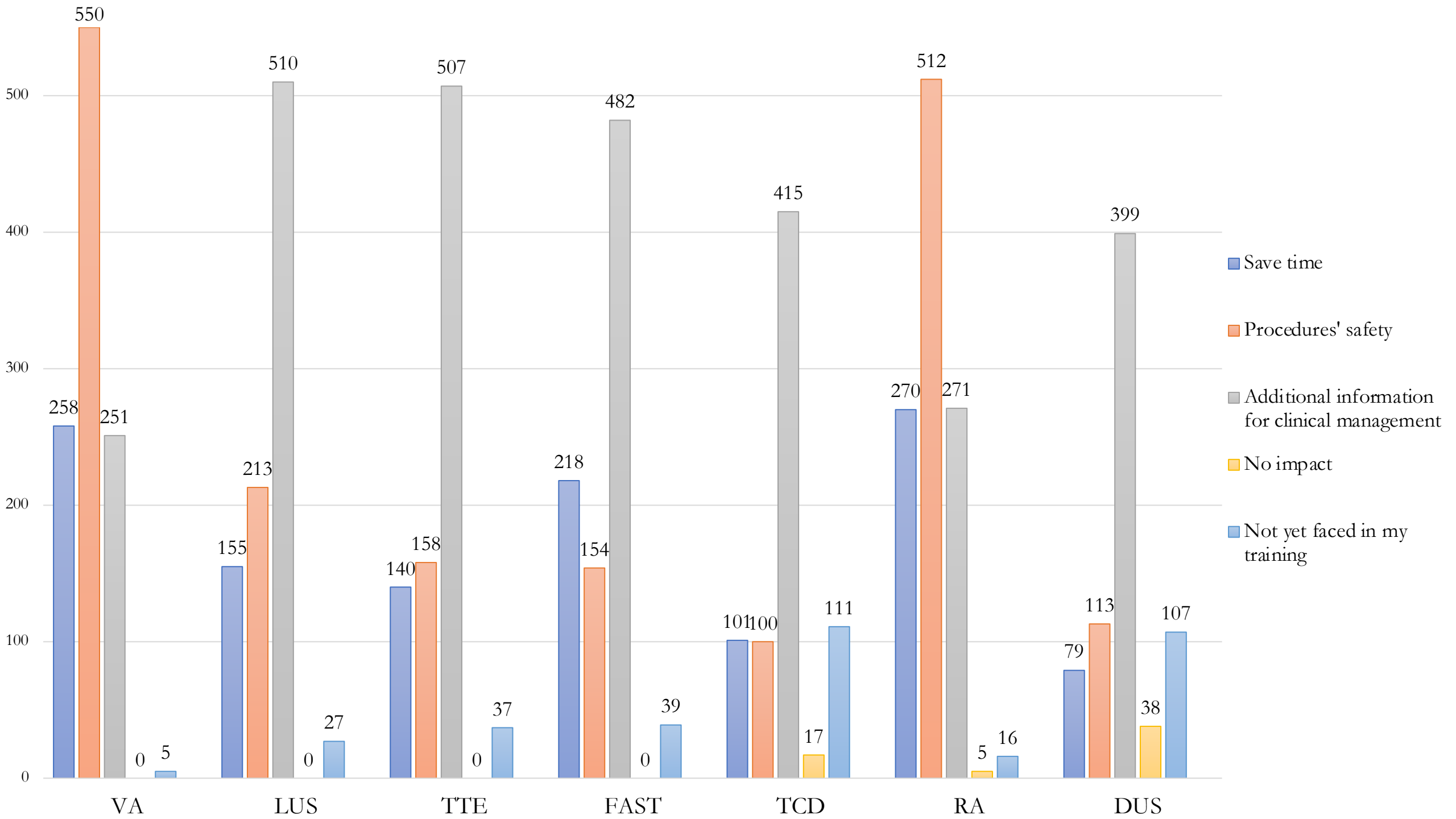

Supplement: Supplementary file 6 — Additional file 6: e-Figure 6. Expected additional value in clinical activity of ultrasound techniques in the residents’ view. VA: vascular access; LUS: lung ultrasound; TTE: transthoracic echocardiography; FAST: focused assessment with sonography in trauma; TCD: transcranial Doppler; RA: regional anaesthesia; DUS: diaphragm ultrasound. [file 12909_2022_3708_MOESM6_ESM.pdf]

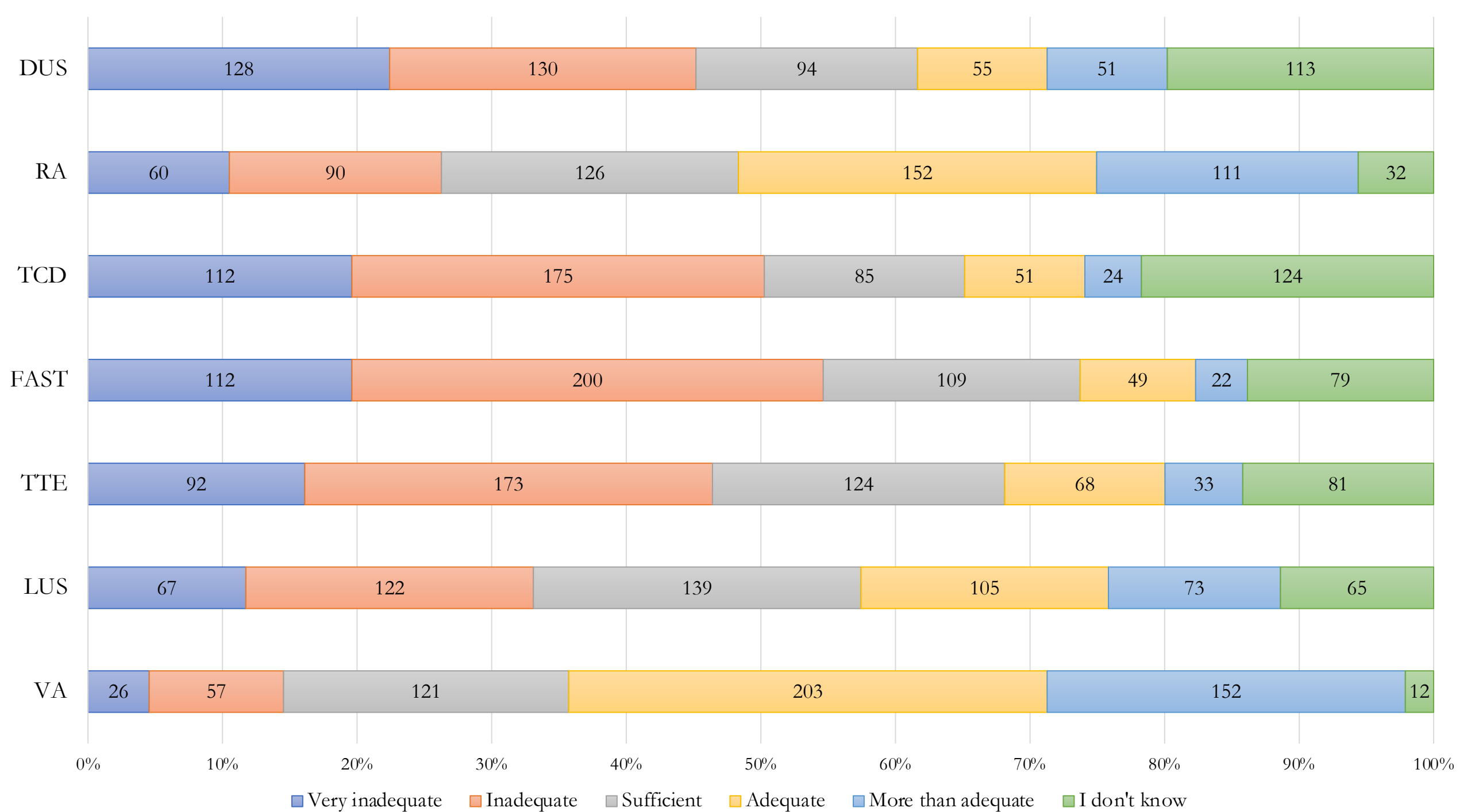

Supplement: Supplementary file 7 — Additional file 7: e-Figure 7. Adequacy of ultrasound training as perceived by residents in the different ultrasound techniques. VA: vascular access; LUS: lung ultrasound; TTE: transthoracic echocardiography; FAST: focused assessment with sonography in trauma; TCD: transcranial Doppler; RA: regional anaesthesia; DUS: diaphragm ultrasound. [file 12909_2022_3708_MOESM7_ESM.pdf]

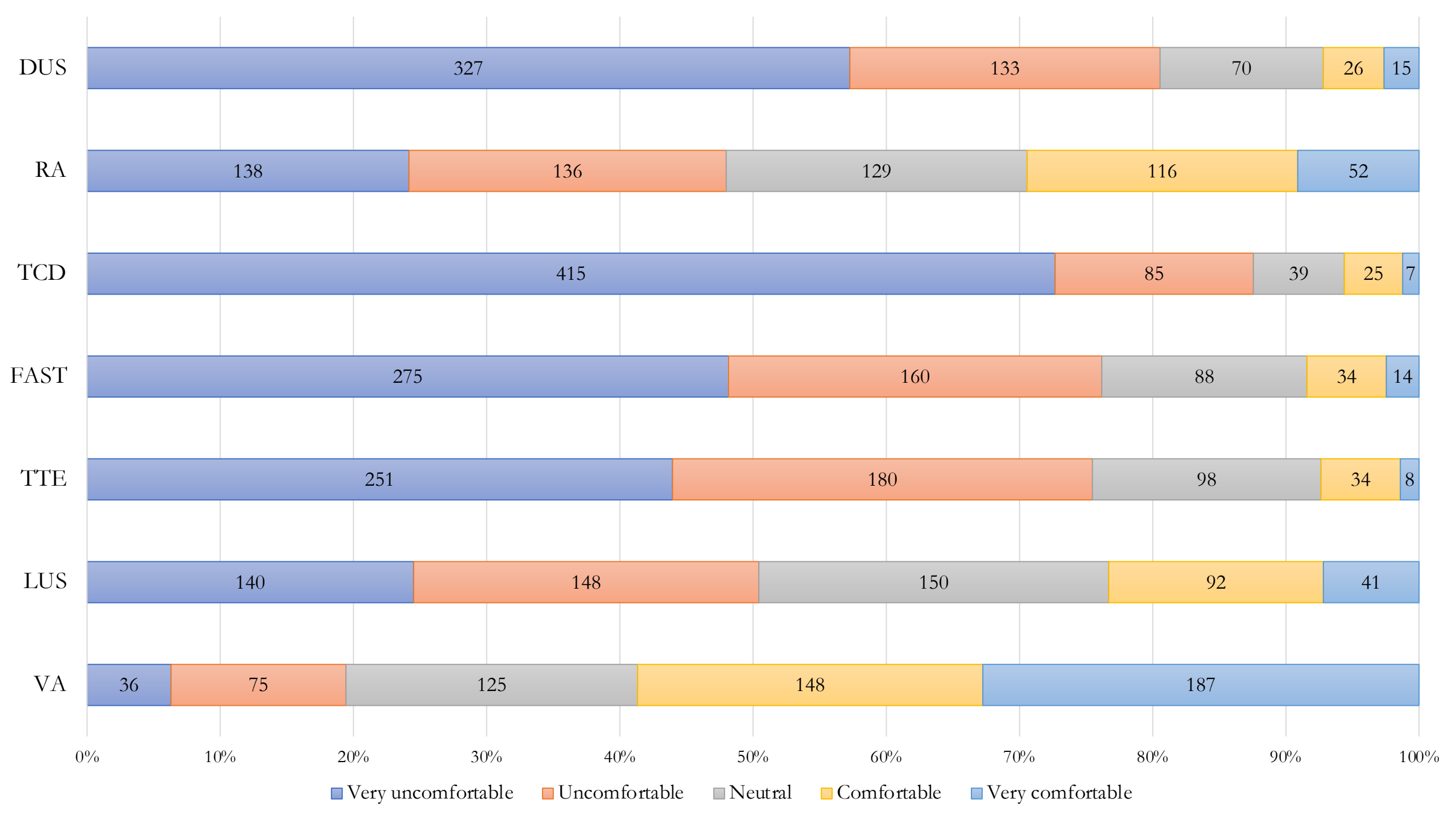

Supplement: Supplementary file 8 — Additional file 8: e-Figure 8. Confidence in performing ultrasound examination and procedures as perceived by residents. VA: vascular access; LUS: lung ultrasound; TTE: transthoracic echocardiography; FAST: focused assessment with sonography in trauma; TCD: transcranial Doppler; RA: regional anaesthesia; DUS: diaphragm ultrasound. [file 12909_2022_3708_MOESM8_ESM.pdf]

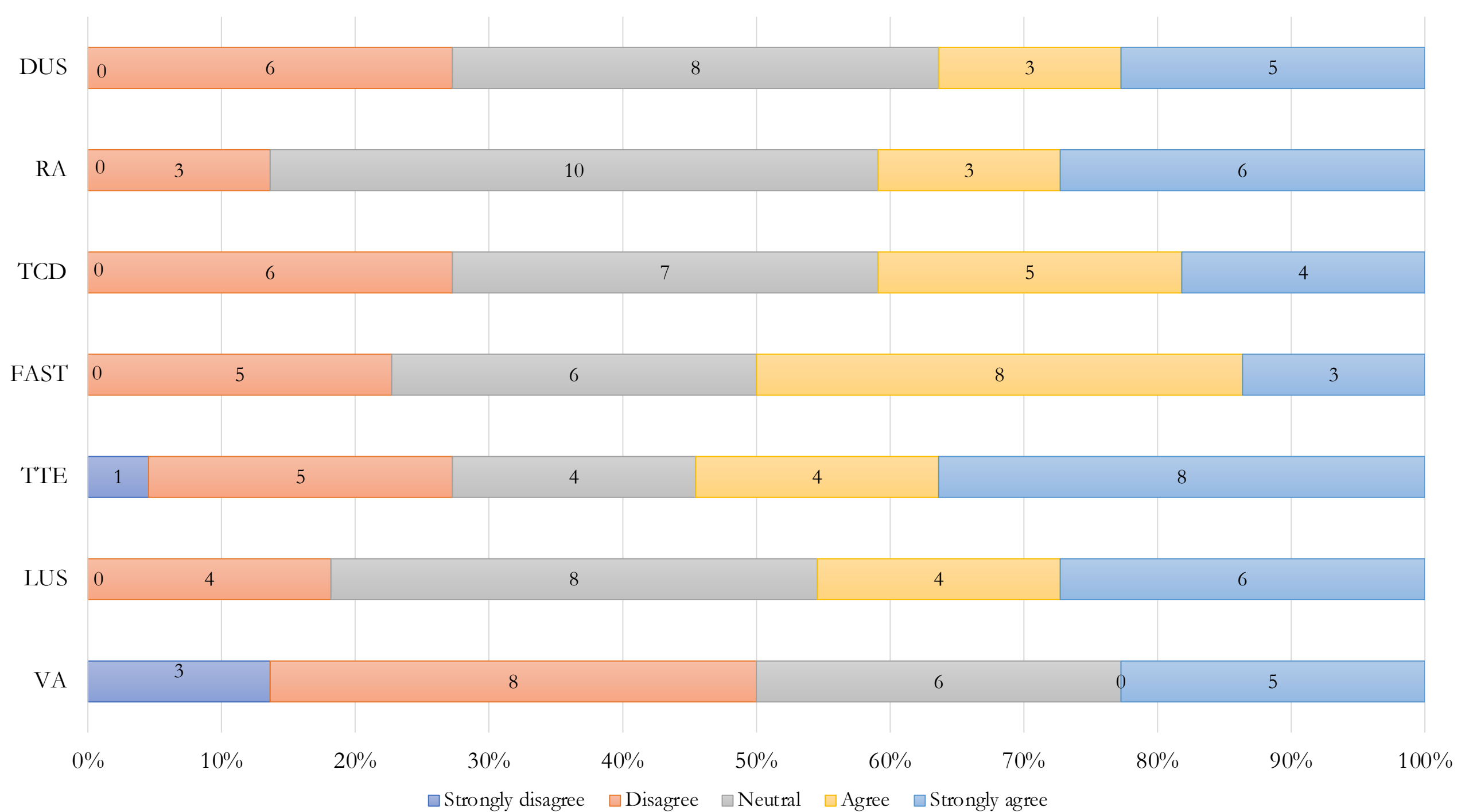

Supplement: Supplementary file 9 — Additional file 9: e-Figure 9. Need of improvement in ultrasound teaching in their own residency school as perceived by directors. VA: vascular access; LUS: lung ultrasound; TTE: transthoracic echocardiography; FAST: focused assessment with sonography in trauma; TCD: transcranial Doppler; RA: regional anaesthesia; DUS: diaphragm ultrasound. [file 12909_2022_3708_MOESM9_ESM.pdf]
