# Supplementary material for: Point-of-care ultrasound training for residents in anaesthesia and critical care: results of a national survey comparing residents and training program directors’ perspectives
Source: BMC Med Educ. 2022 Aug 28;22:647. doi: 10.1186/s12909-022-03708-w (PMC9420188; doi:10.1186/s12909-022-03708-w)
Supplement: Supplementary file 10 — Additional file 10: Appendix 1. Survey sent to directors. [file 12909_2022_3708_MOESM10_ESM.pdf]

1. Indicate which residency school you are from (the answer will NOT be associated to the other results of the survey and will only be used to know which Schools actually filled the survey)

Only one answer is possible

- Ancona - Politecnica delle Marche
- Bari
- Bologna
- Brescia
- Cagliari
- Catania
- Catanzaro
- Chieti - Pescara
- Ferrara
- Firenze
- Foggia
- Genova
- L' Aquila
- Messina
- Milano
- Milano 'Bicocca'
- Milano San Raffaele
- Modena e Reggio Emilia
- Napoli Federico II
- Napoli II Ateneo (SUN) - vanvitelli

- Novara Piemonte Orientale
- Padova
- Palermo
- Parma
- Pavia
- Perugia
- Pisa
- Roma Campus Biomedico
- Roma Cattolica
- Roma La Sapienza
- Roma Sant'Andrea
- Roma Tor Vergata
- Sassari
- Siena
- Torino
- Trieste
- Udine
- Varese 'Insubria'
- Verona
- Humanitas

2. For each POCUS application, indicate which teaching tools are used in your residency school (more than one answer is possible):

	Bedside teaching	Online modules	Frontal lessons	Simulation	Participation to research	None
Vascular access	☐	☐	☐	☐	☐	☐
Lung Ultrasound	☐	☐	☐	☐	☐	☐
Transthoracic Echocardiography	☐	☐	☐	☐	☐	☐
FAST	☐	☐	☐	☐	☐	☐
Transcranial Doppler	☐	☐	☐	☐	☐	☐
Regional Anaesthesia	☐	☐	☐	☐	☐	☐
Diaphragm Ultrasound	☐	☐	☐	☐	☐	☐

3. If there is an ultrasound training, who is the mentor? (more than one answer possible)

- [ ] Self-training
- [ ] A senior resident
- [ ] A consultant physician

4. Indicate the percentage of residents of your school who could attend an extra-curricular ultrasound course with financial support from the school in the last 5 years

Only one answer available

- ( ) 0%
- ( ) 25%
- ( ) 50%
- ( ) 75%
- ( ) 100%

5. How many hours of theoretical training are dedicated to each ultrasound technique in the 5-year residency school?

	0	1-2	3-5	>5
Vascular access	◯	◯	◯	◯
Lung Ultrasound	◯	◯	◯	◯
Transthoracic Echocardiography	◯	◯	◯	◯
FAST	◯	◯	◯	◯
Transcranial Doppler	◯	◯	◯	◯
Regional Anaesthesia	◯	◯	◯	◯
Diaphragm Ultrasound	◯	◯	◯	◯

6. Which is the minimum number of exams required to achieve basic competences in your opinion?

	0	1-10	11-29	>=30
Vascular access	⬭	⬭	⬭	⬭
Lung Ultrasound	⬭	⬭	⬭	⬭
Transthoracic Echocardiography	⬭	⬭	⬭	⬭
FAST	⬭	⬭	⬭	⬭
Transcranial Doppler	⬭	⬭	⬭	⬭
Regional Anaesthesia	⬭	⬭	⬭	⬭
Diaphragm Ultrasound	⬭	⬭	⬭	⬭

7. How important are ultrasound skills in the overall evaluation of the resident?

	1 Not important at all	2	3	4	5 Very important
Vascular access					
Lung Ultrasound					
Transthoracic Echocardiography					
FAST					
Transcranial Doppler					
Regional Anaesthesia					
Diaphragm Ultrasound					

8. Which are the evaluation used in your residency school? More than one answer is possibile

- [ ] None
- [ ] Theoretical examination
- [ ] Bedside evaluation
- [ ] Formal theoretical and practical certification

9. Which other ultrasound technique should be implemented in residency school in your opinion? (open answer)

____

10. Do you think ultrasound training should be improved in your residency school?

	1 - Absolutely disagree	2	3	4	5 - Absolutely agree
Vascular access	○	○	○	○	○
Lung Ultrasound	○	○	○	○	○
Transthoracic Echocardiography	○	○	○	○	○
FAST	○	○	○	○	○
Transcranial Doppler	○	○	○	○	○
Regional Anaesthesia	○	○	○	○	○
Diaphragm Ultrasound	○	○	○	○	○

11. Which is the ultrasound machine availability?

	No ultrasound machine	1 shared with another Unit	1 dedicated machine	>1 dedicated machine
ICU				
Operating Room				
Emergency Department				
Extra-hospital medicine				
Outpatient services (pain therapy, vascular access)				

12. Limiting factors for ultrasound training (more than one answer is possible)

	Limited mentor's time availability	Limited mentor's expertise	Ultrasound machines' availability	Limited resident's time availability	Lack of a standardized curriculum	None
Vascular access	☐	☐	☐	☐	☐	☐
Lung Ultrasound	☐	☐	☐	☐	☐	☐
Transthoracic Echocardiography	☐	☐	☐	☐	☐	☐
FAST	☐	☐	☐	☐	☐	☐
Transcranial Doppler	☐	☐	☐	☐	☐	☐
Regional Anaesthesia	☐	☐	☐	☐	☐	☐
Diaphragm Ultrasound	☐	☐	☐	☐	☐	☐

Questi contenuti non sono creati né avallati da Google.
