# Supplementary material for: Point-of-care ultrasound training for residents in anaesthesia and critical care: results of a national survey comparing residents and training program directors’ perspectives
Source: BMC Med Educ. 2022 Aug 28;22:647. doi: 10.1186/s12909-022-03708-w (PMC9420188; doi:10.1186/s12909-022-03708-w)
Supplement: Supplementary file 11 — Additional file 11: Appendix 2. Survey sent to residents. [file 12909_2022_3708_MOESM11_ESM.pdf]

1. Indicate which residency school you are from (the answer will NOT be associated to the other results of the survey and will only be used to know which Schools actually filled the survey)

Only one answer is possible

- Ancona - Politecnica delle Marche
- Bari
- Bologna
- Brescia
- Cagliari
- Catania
- Catanzaro
- Chieti-Pescara
- Ferrara
- Firenze
- Foggia
- Genova
- L' Aquila
- Messina
- Milano
- Milano 'Bicocca'
- Milano San Raffaele
- Modena e Reggio Emilia
- Napoli Federico II
- Napoli II Ateneo (SUN) - vanvitelli

- Novara Piemonte Orientale
- Padova
- Palermo
- Parma
- Pavia
- Perugia
- Pisa
- Roma Campus Biomedico
- Roma Cattolica
- Roma La Sapienza
- Roma Sant'Andrea
- Roma Tor Vergata
- Sassari
- Siena
- Torino
- Trieste
- Udine
- Varese 'Insubria'
- Verona
- Humanitas

2. Which year of training are you attending?

- First
- Second
- Third
- Fourth
- Fifth

3. For each POCUS application, indicate which teaching tools are used in your residency school (more than one answer is possible):

	Bedside teaching		Online modules	Frontal lessons	Simulation	Participation to research	None
Vascular access	☐	☐	☐	☐	☐	☐	☐
Lung Ultrasound	☐	☐	☐	☐	☐	☐	☐
Transthoracic Echocardiography	☐	☐	☐	☐	☐	☐	☐
FAST	☐	☐	☐	☐	☐	☐	☐
Transcranial Doppler	☐	☐	☐	☐	☐	☐	☐
Regional Anaesthesia	☐	☐	☐	☐	☐	☐	☐
Diaphragm Ultrasound	☐	☐	☐	☐	☐	☐	☐

4. Have you ever attended an extra-curricular ultrasound course?

- Yes, I paid for it
- Yes, with financial support from the school
- No

5. How many hours of theoretical training are dedicated to each ultrasound technique in the 5-year residency school?

	0	1-2	3-5	>5	I don't know, not yet faced in my training
Vascular access					
Lung Ultrasound					
Transthoracic Echocardiography					
FAST					
Transcranial Doppler					
Regional Anaesthesia					
Diaphragm Ultrasound					

6. Which is the minimum number of exams required to achieve basic competences by your residency training program?

	0	1-10	11-29	>=30	I don't know
Vascular access					
Lung Ultrasound					
Transthoracic Echocardiography					
FAST					
Transcranial Doppler					
Regional Anaesthesia					
Diaphragm Ultrasound					

7. How important are considered ultrasound skills in the overall evaluation of the resident by your school?

	1 Not important at all	2	3	4	5 very important	I don't know, not yet faced in my training
Vascular access						
Lung Ultrasound						
Transthoracic Echocardiography						
FAST						
Transcranial Doppler						
Regional Anaesthesia						
Diaphragm Ultrasound						

8. Which are the evaluation used in your residency school? More than one answer is possibile

- [ ] None
- [ ] Theoretical examination
- [ ] Bedside evaluation
- [ ] Formal theoretical and practical certification

9. If there is an ultrasound training, who is the mentor? (more than one answer possible)

- [ ] Self-training
- [ ] A senior resident
- [ ] A consultant physician

10. In your opinion, how important will be in your professional life the following ultrasound skills

	1 Not important at all	2	3	4	5 Very important	I don't know, not yet faced in my training
Vascular access						
Lung Ultrasound						
Transthoracic Echocardiography						
FAST						
Transcranial Doppler						
Regional Anaesthesia						
Diaphragm Ultrasound						

11. How will ultrasound skill impact on your work? More than one answer possible

	Save time	Procedures' safety	Additional clinical information	No impact	I don't know, not yet faced in my training
Vascular access	☐	☐	☐	☐	☐
Lung Ultrasound	☐	☐	☐	☐	☐
Transthoracic Echocardiography	☐	☐	☐	☐	☐
FAST	☐	☐	☐	☐	☐
Transcranial Doppler	☐	☐	☐	☐	☐
Regional Anaesthesia	☐	☐	☐	☐	☐
Diaphragm Ultrasound	☐	☐	☐	☐	☐

12. Which other ultrasound technique should be implemented in residency school in your opinion? (open answer)

____

13. In the relevance accorded to ultrasound adequate in your school?

	Very inadequate	Inadequate	Sufficient	Adequate	More than adequate	I don't know, not yet faced in my training
Vascular access						
Lung Ultrasound						
Transthoracic Echocardiography						
FAST						
Transcranial Doppler						
Regional Anaesthesia						
Diaphragm Ultrasound						

14. Do you feel comfortable in taking clinical decisions or performing ultrasound guided procedures?

	1 Not at all	2	3	4	5 Yes definitely
Vascular access					
Lung Ultrasound					
Transthoracic Echocardiography					
FAST					
Transcranial Doppler					
Regional Anaesthesia					
Diaphragm Ultrasound					

15. Which is the ultrasound machine availability?

	No ultrasound machine	1 shared with another Unit	1 dedicated machine	>1 dedicated machine	
ICU					
Operating Room					
Emergency Department					
Extra-hospital medicine					
Outpatient services (pain therapy, vascular access)					

16. Limiting factors for ultrasound training (more than one answer is possible)

	Limited mentor's time availability	Limited mentor's expertise	Ultrasound machines' availability	Limited resident's time availability	Lack of a standardized curriculum	I don't know, I haven't faced yet in my training
Vascular access	☐	☐	☐	☐	☐	☐
Lung Ultrasound	☐	☐	☐	☐	☐	☐
Transthoracic Echocardiography	☐	☐	☐	☐	☐	☐
FAST	☐	☐	☐	☐	☐	☐
Transcranial Doppler	☐	☐	☐	☐	☐	☐
Regional Anaesthesia	☐	☐	☐	☐	☐	☐
Diaphragm Ultrasound	☐	☐	☐	☐	☐	☐

17. Do you use additional teaching tools for ultrasound?

- [ ] Online tutorial, web-based lectures, videos associated to articles
- [ ] Books/scientific literature
- [ ] Extra-curricular courses
- [ ] Other
- [ ] No

Questi contenuti non sono creati né avallati da Google.

Google Moduli
