# Supplementary material for: Point-of-care ultrasound training for residents in anaesthesia and critical care: results of a national survey comparing residents and training program directors’ perspectives
Source: BMC Med Educ. 2022 Aug 28;22:647. doi: 10.1186/s12909-022-03708-w (PMC9420188; doi:10.1186/s12909-022-03708-w)
Supplement: Supplementary file 12 — Additional file 12: e-Table 1. Availability of ultrasound machines in different clinical contexts according to directors and residents. [file 12909_2022_3708_MOESM12_ESM.docx]

**e-Table 1** Availability of ultrasound machines in different clinical contexts according to directors and residents.

|  | **Directors**  **N=22** | | | | **Residents**  **N=571** | | | | | ***P* value** |
| --- | --- | --- | --- | --- | --- | --- | --- | --- | --- | --- |
|  | No machines | 1 shared | 1 dedicated | >1 dedicated | No machines | 1 shared | 1 dedicated | >1 dedicated | I don’t know |  |
| Intensive Care Unit | 0  (0.0) | 0  (0.0) | 9  (40.9) | 13  (59.1) | 0  (0.0) | 45  (8.5) | 271  (51.4) | 209  (39.7) | 44 | 0.146 |
| Operating Room | 0  (0.0) | 2  (9.0) | 11  (50.0) | 9  (40.9) | 24  (4.4) | 141  (25.8) | 245  (44.8) | 137  (43.0) | 24 | 0.154 |
| Emergency Department | 1  (4.5) | 3  (13.6) | 12  (54.5) | 6  (27.3) | 22  (6.9) | 47  (14.7) | 204  (63.9) | 46  (14.4) | 252 | 0.445 |
| Pre-hospital medicine | 12  (54.5) | 5  (22.7) | 2  (9.0) | 3  (13.6) | 67  (48.9) | 10  (7.3) | 46  (33.6) | 14  (10.2) | 434 | **0.020** |
| Outpatient Units (pain therapy, vascular access) | 0  (0.0) | 5  (22.7) | 13  (59.1) | 4  (18.2) | 50  (16.2) | 86  (27.9) | 139  (45.1) | 33  (10.7) | 263 | **0.079** |

Data are displayed as values (percentage). In bold: statistically significant p values; for residents. Fisher exact and percentages were computed excluding those answering: “I don’t know”.
